# Training status affects between-protocols differences in the assessment of maximal aerobic velocity

**DOI:** 10.1007/s00421-021-04763-9

**Published:** 2021-07-28

**Authors:** Andrea Riboli, Susanna Rampichini, Emiliano Cè, Eloisa Limonta, Marta Borrelli, Giuseppe Coratella, Fabio Esposito

**Affiliations:** 1 Department of Biomedical Sciences for Health (SCIBIS), University of Milan, Via G. Colombo 71, 20133 Milan, Italy; 2 IRCCS, Istituto Ortopedico Galeazzi, Via R. Galeazzi 4, 20161 Milan, Italy

**Keywords:** $\dot{V}$O_2_ kinetics, Maximal aerobic power, Maximum oxygen uptake, Incremental test, Running velocity, Aerobic capacity

## Abstract

**Purpose:**

Continuous incremental protocols (CP) may misestimate the maximum aerobic velocity (V_max_) due to increases in running speed faster than cardiorespiratory/metabolic adjustments. A higher aerobic capacity may mitigate this issue due to faster pulmonary oxygen uptake ($\dot{V}$O_2_) kinetics. Therefore, this study aimed to compare three different protocols to assess V_max_ in athletes with higher or lower training status.

**Methods:**

Sixteen well-trained runners were classified according to higher (HI) or lower (LO) $\dot{V}$O_2max_ $\dot{V}$O_2_-kinetics was calculated across four 5-min running bouts at 10 km·h^−1^. Two CPs [1 km·h^−1^ per min (CP1) and 1 km·h^−1^ every 2-min (CP2)] were performed to determine V_max_ $\dot{V}$O_2max_, lactate-threshold and submaximal $\dot{V}$O_2_/velocity relationship. Results were compared to the discontinuous incremental protocol (DP).

**Results:**

V_max_, $\dot{V}$O_2max_, $\dot{V}$CO_2_ and VE were higher [(*P* < 0.05,(ES:0.22/2.59)] in HI than in LO. $\dot{V}$O_2_-kinetics was faster [*P* < 0.05,(ES:-2.74/ − 1.76)] in HI than in LO. $\dot{V}$O_2_/velocity slope was lower in HI than in LO [(*P* < 0.05,(ES:-1.63/ − 0.18)]. V_max_ and $\dot{V}$O_2_/velocity slope were CP1 > CP2 = DP for HI and CP1 > CP2 > DP for LO. A lower [*P* < 0.05,(ES:0.53/0.75)] V_max_-difference for both CP1 and CP2 *vs* DP was found in HI than in LO. V_max_-differences in CP1 *vs* DP showed a *large* inverse correlation with V_max_, $\dot{V}$O_2max_ and lactate-threshold and a *very large* correlation with $\dot{V}$O_2_-kinetics.

**Conclusions:**

Higher aerobic training status witnessed by faster $\dot{V}$O_2_ kinetics led to lower between-protocol V_max_ differences, particularly between CP2 *vs* DP. Faster kinetics may minimize the mismatch issues between metabolic and mechanical power that may occur in CP. This should be considered for exercise prescription at different percentages of V_max_.

## Introduction

A successful aerobic performance depends on several physiological, biomechanical, and psychological factors (Bentley et al. 2007; Coyle 1995). Among physiological aspects, a high maximum pulmonary oxygen uptake ($\dot{V}$O_2max_), the ability to maintain a long time to exhaustion at V̇O_2max_, a faster $\dot{V}$O_2_-transition from rest to steady-condition ($\dot{V}$O_2_ kinetics), a higher lactate threshold and a low O_2_ cost of running are the main parameters of aerobic performance (Poole and Richardson 1997; Coyle 1995; Poole and Jones 2012).

Also the maximum aerobic velocity (V_max_), defined as the minimum velocity capable to elicit $\dot{V}$O_2max_ when considering only the completion of the primary phase of $\dot{V}$O_2_-on kinetics (Ferretti 2015), is reported as a strong marker of running performance (Bentley et al. 2007) and it integrates both metabolic and biomechanical aspects of running into a single factor (Buchheit and Laursen 2013). In elite aerobic athletes, a higher V_max_ reflects a greater capacity to utilize the aerobic metabolic pathways across several sports (Noakes 1988; Pedro et al. 2013; Ziogas et al. 2011; Rampinini et al. 2007).

$\dot{V}$O_2max_ and V_max_ are generally determined using different incremental running protocols (Kuipers et al. 2003; Riboli et al. 2017), among which continuous or discontinuous tests that may vary in work rate increments and stage duration (Billat et al. 1996; Kuipers et al. 2003; Riboli et al. 2017). Discontinuous incremental protocols (DP) are characterized by constant work rates interspersed by resting periods (Duncan et al. 1997; Riboli et al. 2017). DP permits to reach an equilibrium between the cardiorespiratory and metabolic systems and the work rate when lasting at least three minutes to achieve a steady-state condition (Poole and Jones 2012). However, the long overall duration of DP would markedly lengthen the whole testing phase, thus affecting the possibility to test several athletes within one single session, as often required in sports practice. Conversely, incremental continuous protocols (CP) last short overall duration and they have been shown as a valid and reliable method to determine $\dot{V}$O_2max_ despite the submaximal physiological adjustments cannot be reached as in DP due to increments in work rate faster than cardiorespiratory and metabolic adjustments (Riboli et al. 2017, 2021). Despite in some intermittent protocols with very low workload *vs* recovery ratio $\dot{V}$O_2max_ may not be reached (Vinetti et al. 2017), previous studies using CP and DP showed that $\dot{V}$O_2max_ was found to be independent from the protocol adopted (Kuipers et al. 2003; Riboli et al. 2017, 2021). Conversely, testing protocols with shorter stage duration may lead to higher V_max_ (Riboli et al. 2017; Kuipers et al. 2003; Adami et al. 2013). Given that V_max_ is currently utilized to prescribe or monitor training routines (Buchheit and Laursen 2013; Riboli et al. 2021), a precise V_max_ assessment may allow coaches to manipulate accurately the physiological load during running exercises as a percentage of V_max_ (Buchheit and Laursen 2013; Riboli et al. 2021). For instance, 90–110% of V_max_ are suggested for long-interval exercises, 110–130% V_max_ for short-intervals exercises, 130–160% V_max_ for repeated sprint training and > 160% V_max_ for sprint interval training (Buchheit and Laursen 2013). Therefore, a precise V_max_ assessment should be carefully taken into account for athletes’ testing and training prescription (Riboli et al. 2017; Bentley et al. 2007).

Athletes with a high aerobic capacity (HI), such as long- and middle-distance runners, are qualified by greater physiological characteristics in terms of high $\dot{V}$O_2max_ and fast $\dot{V}$O_2_ kinetics (Coyle 1995) than in individuals with lower aerobic capacity (LO). A high $\dot{V}$O_2max_ represents, indeed, a pronounced maximal pulmonary, cardiovascular, metabolic and muscular capacity to uptake, transport and utilize O_2_ (Poole and Richardson 1997). Moreover, rapid $\dot{V}$O_2_ kinetics may lead to a smaller O_2_ deficit and a reduced intracellular perturbation, thus reflecting greater exercise tolerance (Poole and Jones 2012; Dupont et al. 2005) and endurance performance (Poole and Jones 2012). These characteristics in HI may therefore lower or even minimize the misestimating issue that may occur in CP because of their faster $\dot{V}$O_2_ kinetics.

With this in mind, the present study aimed to investigate how aerobic training status may affect V_max_ assessment during CPs *vs* DP in two groups of athletes, characterized by different aerobic training conditions. Should HI in the investigated group demonstrate faster $\dot{V}$O_2_ kinetics due to their greater ability of the cardiorespiratory and metabolic systems to adjust to continuous increases in work rate typical of CP, the V_max_ misestimating issue may be minimized, when comparing their CPs to DP results.

## Materials and methods

### Participants

Sixteen well-trained middle and long-distance runners (age: 22.1 ± 1.8 years; stature: 1.75 ± 0.05 m; body mass: 70.3.7 ± 3.7 kg; mean ± standard deviation) volunteered to participate in the study and were classified into two groups, according to their higher (HI) or lower (LO) $\dot{V}$O_2max_ and the International Physical Activity Questionnaire (IPAQ). All participants met the following criteria: (a) more than four years of systematic training and (b) no injuries in the last year. The ethics committee of the local University approved the study (protocol #102/14) which was performed in accordance with the principles of the Declaration of Helsinki (1964 and updates). All participants gave their written consent after a full explanation of the purpose of the study and the experimental design.

### Study design

To test the current hypothesis, two incremental continuous protocols with different stage durations (CP) were performed and compared to a discontinuous incremental protocol (DP). The present study spanned over a maximum of 3 weeks. The participants reported to the laboratory five times, separated by at least 72 h. During the first visit, they were familiarized with the experimental procedures. During the second session, they performed a continuous incremental protocol (1 km⋅h^−1^ per minute) to determine $\dot{V}$O_2max_ and to complete the IPAQ. Within the remaining three sessions, the participants randomly underwent the three experimental conditions (two continuous and one discontinuous incremental protocols). Within each testing-session, an initial 5-min submaximal bout at 10 km⋅h^−1^ was modelled to determine the on-transient $\dot{V}$O_2_ kinetics. Participants were instructed to avoid any form of strenuous exercise in the three days before each session. In addition, they were asked to have their last standardized meal at least three hours before each session. Finally, they were requested to abstain from ergogenic and caffeinated beverages before testing.

Participants were split subsequently into two groups, according to their $\dot{V}$O_2max_ normalized per body mass (ml·kg^−1^·min^−1^) and their training routines (i.e., *n* of training sessions per week). The first HI group was characterized by a higher $\dot{V}$O_2max_ and more than five training sessions per week. The second LO group was characterized by a lower $\dot{V}$O_2max_ and no more than three training sessions per week.

### Experimental procedures

All tests were conducted approximately at the same time of the day in a climate-controlled laboratory (constant temperature of 20 ± 1 °C and relative humidity of 50 ± 5%). All tests were carried out on a treadmill ergometer (RAM s.r.l., mod. 770 S, Padova, Italy) with a 1% positive slope. Blood lactate concentration (BLa^−^) was assessed by a spectrophotometric system (Lactate Pro LT-1710, Arkray, Kyoto, Japan). The lactate analyzer was calibrated before each protocol to guarantee consistent data. $\dot{V}$O_2max_, expiratory ventilation, carbon dioxide production and respiratory exchange ratio were measured during each protocol by a gas analyzer cart (Cosmed, mod. Quark *b*2, Rome, Italy). The device was calibrated before each test with gas mixtures of known concentration (O_2_ 16%, CO_2_ 5%, balance N_2_). Heart rate was monitored continuously using a heart rate monitor (Polar Electro Oy, mod. S810i, Kempele, Finland). Arterial O_2_ saturation was determined by a finger-tip infrared oxymeter (NONIN Medical, mod. 3011, Minneapolis, MN). At the end of the test, the rate of perceived exertion (RPE) was determined using the 6–20 Borg scale for general, respiratory and muscular fatigue. The participants were strongly encouraged by the operators to perform each test up to their maximum exercise capacity.

Continuous Incremental Protocol 1 (CP1). After 5 min of baseline measurements, while standing on the treadmill, the participants warmed up at 10 km⋅h^−1^ for 5 min. Then, the running speed was increased progressively by 1 km⋅h^−1^ per minute until volitional exhaustion. BLa^−^ was measured at baseline, at the end of each stage and after 1, 3 and 5 min of passive recovery. The achievement of V̇O_2max_ was identified as the plateauing of $\dot{V}$O_2_ (< 2.1 ml·kg^−1^·min^−1^ increase**)** despite an increase in workload (Poole and Richardson 1997). If the above-stated criterion and/or secondary criteria to establish $\dot{V}$O_2max_ (Poole et al. 2008) were not fulfilled, the participants were asked to perform a further constant-speed test equal or higher than the highest speed achieved at the end of the incremental test, as strongly recommended (Rossiter et al. 2006). $\dot{V}$O_2_, carbon dioxide production, expiratory ventilation, O_2_ saturation and respiratory exchange ratio were averaged during the last 30 s of each step at submaximal workload and over the last 30 s before exhaustion. V_max_ was determined as the minimal running velocity that elicited $\dot{V}$O_2max_ over a period of 30 s (Billat et al. 1996). If a stage could not be completed, the V_max_ was calculated according to a previously published equation (Kuipers et al. 2003) [V_max_ = V_completed_ + t/T x speed increment], in which V_completed_ is the running speed of the last stage that was completed, t the number of seconds that the uncompleted running stage could be sustained, T the number of seconds required to complete the stage, and speed increment is the speed load increment in km⋅h^−1^.

Continuous Incremental Protocol 2 (CP2). CP2 followed the same experimental procedures as CP1, but with the increases in treadmill running speed of 1 km⋅h^−1^ every two minutes. As for CP1, $\dot{V}$O_2_, carbon dioxide production, expiratory ventilation, O_2_ saturation, and respiratory exchange ratio were averaged during the last 30 s of each step at submaximal workload and over the last 30 s before exhaustion. V_max_ was determined as the minimal running velocity that elicited $\dot{V}$O_2max_ over a period of 30 s (Billat et al. 1996).

Discontinuous Incremental Protocol (DP). DP protocol involved five workloads of 4 min each, interspersed by at least 5 min of recovery (Bernard et al. 2000). The optimal stage duration suggested for DPs is still questioned (Bernard et al. 2000). Although some authors suggested that it should be around 6–8 min (Bernard et al. 2000), it was criticized that relatively long stage duration could result in premature fatigue and suggested that 4–6 min could be suitable for this purpose (Bentley et al. 2007; Kuipers et al. 2003; Bernard et al. 2000). Since shorter test duration is strongly advocated during in-field practice, a 4-min stage duration was used here.

Baseline measurements were recorded with the participants standing on the treadmill. The first two workloads were set at 8 and 10 km·h^−1^ for all participants. The following three workloads were tailored for each participant according to the individual cardiorespiratory responses to the first two workloads and considering the theoretical maximum heart-rate determined (Bernard et al. 2000). Firstly, based on the $\dot{V}$O_2_ and the heart-rate recorded during the first two stages, a sub-maximal linear regression was determined up to the predicted peak heart rate, to predict the speed corresponding to possible exhaustion (Bernard et al. 2000). Then, the third, the fourth and the fifth workloads corresponded to approximately 80%, 90% and 105% of the predicted peak workload, respectively. The fourth and the fifth workloads were recalculated using the heart-rate and $\dot{V}$O_2_ recorded during the third and the fourth stage, respectively. The last stage was tailored to let the participants maintain the task for at least four minutes (Bernard et al. 2000). The blood lactate concentration was measured at baseline and after 1, 3 and 5 min of passive recovery for each workload, and the peak blood lactate was inserted into the data analysis. $\dot{V}$O_2_, carbon dioxide production, expiratory ventilation, O_2_ saturation and respiratory exchange ratio were determined as the average value of the last (fourth) minute during each workload (Poole and Richardson 1997). V_max_ was extrapolated from the regression analysis equation of $\dot{V}$O_2_ as a function of running velocity at submaximal workloads below the lactate threshold (Bernard et al. 2000; Riboli et al. 2017).

### ***Lactate threshold, ***$\dot{V}$O_***2***_***/Velocity slope at submaximal exercise and ***$\dot{V}$O_***2***_*** kinetics***

Lactate threshold was determined by the D_MAX_ method, according to which it was identified as the point on the third-order polynomial curve that yielded the maximal perpendicular distance to the straight line formed by the two end data points (Riboli et al. 2019). Similar to the previous study, lactate threshold calculated from CP1 was utilized to limit the range of exercise during which the $\dot{V}$O_2_ *vs* running velocity relationship at submaximal exercise was considered (Riboli et al. 2017).

$\dot{V}$O_2_/Velocity slope: the $\dot{V}$O_2_/Velocity slope was calculated as the regression analysis of the $\dot{V}$O_2_ *vs* velocity relationship at submaximal workloads below lactate threshold for CP1, CP2 and DP (Anderson 1996; Fletcher et al. 2009).

$\dot{V}$O_2_ kinetics. The on-transient $\dot{V}$O_2_ kinetics were modelled after four different bouts of 5-min submaximal exercise (10 km·h^−1^, moderate intensity, below lactate threshold) to avoid any effect of the slow component phenomenon (Jones et al. 2011). The influence of the inter-breath noise was reduced averaging the results of four identical tests in each participant (Lamarra et al. 1987). Each abnormal breath (e.g., different from the mean of the adjacent four data point by more than three times the standard-deviation of those four point, were excluded (Dupont et al. 2005). To increase the time resolution the breath-by-breath $\dot{V}$O_2_ data were subsequently linearly interpolated, and the four data sets were averaged together to produce a single response for each subject. This procedure was previously established to reduce the noise of the $\dot{V}$O_2_ signal and to provide the highest confident results (Poole and Jones 2012). The on-transient of the $\dot{V}$O_2_ kinetics were modelled as previously proposed (Barstow and Mole 1991). The time-delay of the cardiodynamic-phase and the time-constant of the primary-phase (i.e., the time to reach 63% of the $\dot{V}$O_2_ steady-state of the $\dot{V}$O_2_ kinetics were calculated to determine the amplitude of $\dot{V}$O_2_ from baseline to steady-state (Poole and Jones 2012). Then, the mean response time of the on-transition $\dot{V}$O_2_ kinetics as the sum of time-delay and time-constant was calculated. The time-delay, the time-constant and the mean response time were thereafter inserted into data analysis.

### Statistical analysis

Statistical analysis was performed using a statistical software package (Sigma Plot for Windows, v 12.5, Systat Software Inc., San Jose, CA, USA). To check the normal distribution of the sampling, a Kolgomorov-Smirnov test was applied. A one-way analysis of variance (ANOVA) for repeated measures was used also to assess significant differences in V_max_, $\dot{V}$O_2max_, carbon dioxide production, respiratory exchange ratio, arterial O_2_ saturation, heart-rate, expiratory ventilation, blood lactate concentration, $\dot{V}$O_2_/Velocity slope (for both slope and intercept of the submaximal regression analysis equation), $\dot{V}$O_2_ kinetics (time-delay, time-constant and mean-response time), general-, muscular-, and respiratory-RPE between CP1, CP2 and DP. For all pairwise multiple comparisons, a post-hoc Shapiro–Wilk test was applied. A regression analysis was used to assess the relationship between $\dot{V}$O_2_ and running velocity at submaximal exercise. The magnitude of the changes was assessed using Cohen’s standardized effect size (ES) with 95% confidence intervals (95% CI). Effect size with 95% CI was calculated and interpreted as follows: < 0.20: *trivial*; 0.20–0.59: *small;* 0.60–1.19: *moderate;* 1.20–1.99: *large*; ≥ 2.00: *very large* (Hopkins et al. 2009). Pearson’s product moment and 95% CI were utilized to assess the relationship among protocols for V_max_. The correlation coefficients were interpreted as follows: *r* < 0.1 *trivial*; 0.1 ≤ *r* < 0.3 *small*; 0.3 ≤ *r* < 0.5 *moderate*; 0.5 ≤ *r* < 0.7 *large*; 0.7 ≤ *r* < 0.9 *very large*; 0.9 ≤ *r* < 1 *nearly perfect*. Statistical significance was set at an α level of 0.05. Unless otherwise stated, all values are presented as mean ± standard deviation (SD).

## Results

### Between-groups differences

As shown in Table 1, V_max_ [*P* < 0.001, (ES:1.85/2.59)], $\dot{V}$O_2max_ [*P* < 0.001, (ES:0.85/1.07)], VCO_2_ [*P* < 0.001, (ES:0.22/0.61)] and VE [*P* < 0.001, (ES:0.57/0.82] were *small* to *very largely* higher in HI than LO within-each protocol (CP1, CP2 and DP) (Table 1). No between-groups differences (*P* > 0.05) in respiratory exchange ratio, arterial O_2_ saturation, heart rate, BLa^−^_peak_, general-, respiratory-, and muscular-RPE were found.

**Table 1:** Cardiorespiratory, metabolic, and perceptual variables at maximum exercise for HI and LO groups. Mean (SD)

	HI			LO		
	CP1	CP2	DP	CP1	CP2	DP
V_max_ (km·h^−1^)	22.1 (1.2)^*^	19.9 (1.2)^*, **^	19.5 (1.3)	19.1 (1.8)^*^, ^***^	17.2 (1.4) ^*,**,***^	16.2 (1.1)^***^
$\dot{V}$O_2_ (ml·min^−1^)	4169.6 (478.9)	4132.8 (134.2)	4158.8 (473.5)	3912.0 (442.6)^***^	3907.8 (356.4) ^***^	3895.3 (424.9) ^§^
$\dot{V}$O_2_ (ml·kg·min^−1^)	59.2 (5.2)	58.7 (5.4)	59.1 (5.2)	54.6 (4.8)^***^	54.4 (4.1) ^***^	54.5(2.5)^***^
$\dot{V}$CO_2_ (ml·min^−1^)	4581.9 (510.4)	4492.8 (110.8)	4665.2 (442.0)	4465.8 (494.7)^***^	4366.4 (473.0) ^***^	4371.7 (463.0) ^***^
RER	1.10 (0.09)	1.09 (0.03)	1.13 (0.04)	1.13 (0.06)	1.11 (0.06)	1.12 (0.06)
SaO_2_ (%)	89.8 (2.7)	89.6 (1.8)	89.8 (2.7)	91.0 (1.7)	90.6 (2.7)	90.1 (2.7)
***f***_**H**_ (beats·min^−1^)	188.0 (10.0)	188 (10.0)	186.0 (7.0)	189.0 (1.0)	188.0 (5.0)	187.0 (7.0)
$\dot{V}$E (l·min^−1^)	166.9 (19.4)	164.1 (4.2)	163.3 (10.9)	155.1 (19.4)^***^	156.2 (14.9) ^***^	155.4 (7.0) ^***^
BLa^−^_peak_ (mM)	13.0 (4.0)	11.4 (2.3)	12.5 (2.1)	11.4 (1.3)	11.9 (1.0)	11.8 (0.8)
General RPE (au)	18.2 (1.2)	17.9 (1.3)	18.0 (1.3)	18.1 (2.1)	18.3 (1.5)	18.9 (1.2)
Respiratory RPE (au)	18.5 (1.2)	17.7 (1.4)	17.7 (1.4)	17.6 (3.1)	17.8 (1.7)	18.8 (1.0)
Muscular RPE (au)	17.4 (1.5)	17.9 (1.8)	18.4 (1.5)	17.8 (1.7)	17.9 (2.6)	18.1 (1.9)

*V*_*max*_ velocity associated with maximum oxygen uptake; $\dot{V}$*O*_*2*_ oxygen uptake; $\dot{V}$*CO*_*2*_ carbon dioxide production; *RER* respiratory exchange ratio; *SaO*_*2*_ arterial O_2_ saturation; *f*_H_ heart rate frequency; $\dot{V}$E_,_ expiratory ventilation; *BLa*^*−*^_*peak*_ peak blood lactate concentration; and rate of perceived exertion (RPE) at general, respiratory, and muscular level. Variables were determined at maximum exercise in the three testing conditions (CP1, continuous ramp 1; CP2, continuous ramp 2; DP, discontinuous protocol).

^*^P < 0.05 *vs* DP; ^**^P < 0.05 *vs* CP1; ^***^P < 0.05 vs HI

**Fig. 1:**
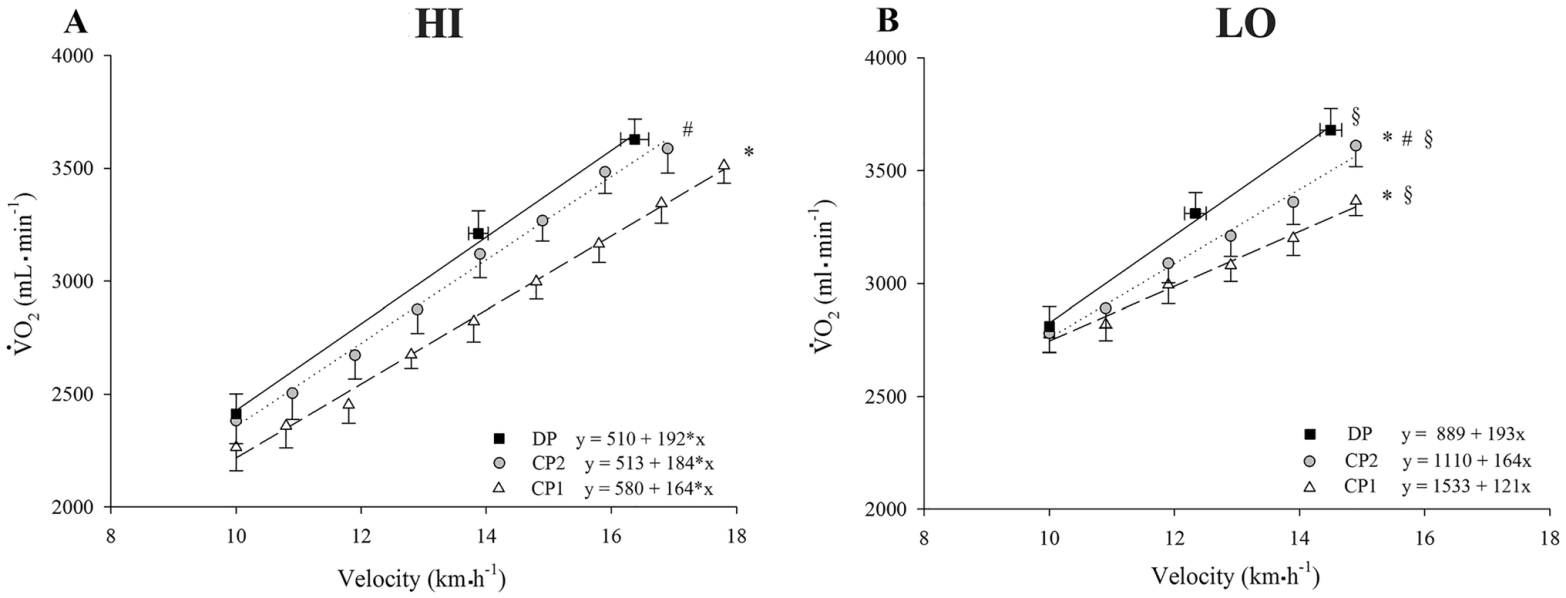
The $\dot{V}$O_2_ as a function of running velocity at submaximal work rates (below the velocity corresponding to the lactate threshold calculated in CP1 condition) for both HI and LO. The solid, dashed and dotted lines represent the regression lines for the discontinuous (DP), continuous protocol with 1 km·h^−1^ increment per minute (CP1) and 2 km·h^−1^ increment every 2 min (CP2), respectively. Panel **A** and **B** show HIGH and LOW group, respectively. Regression equations (*y* = *a · bx*) and correlation coefficients are also reported. *P < 0.05 *vs* DP for slope and intercept of the regression equation, ^#^*P* < 0.05 *vs* CP1 for slope of the regression equation, ^§^*P* < 0.05 *vs* HI for the intercept of the regression equation

The lactate threshold calculated in CP1 was *moderately* [ES:1.99(CI:0.79/3.19)] higher (*P* < 0.001) in HI [17.8(1.1)] than LO [16.1(0.3)]. Overall, the submaximal regression analysis of $\dot{V}$O_2_/velocity relationship for CP1, CP2 and DP was less steep (*P* < 0.05) in HI than LO (Fig. 1); in details, the intercept of the submaximal regression analysis in $\dot{V}$O_2_/velocity relationship ($\dot{V}$O_2_/velocity intercept) was *moderately* to *largely* (ES:-0.86/-1.63) lower (*P* < 0.05) in HI than LO within-each protocol (CP1, CP2 and DP). The slope of the submaximal regression analysis in $\dot{V}$O_2_/velocity relationship ($\dot{V}$O_2_/velocity slope) showed *trivial* to *moderate* (ES:-0.18/0.83) not significant (*P* > 0.05) differences between HI and LO in CP1, CP2 and DP.

**Fig. 2:**
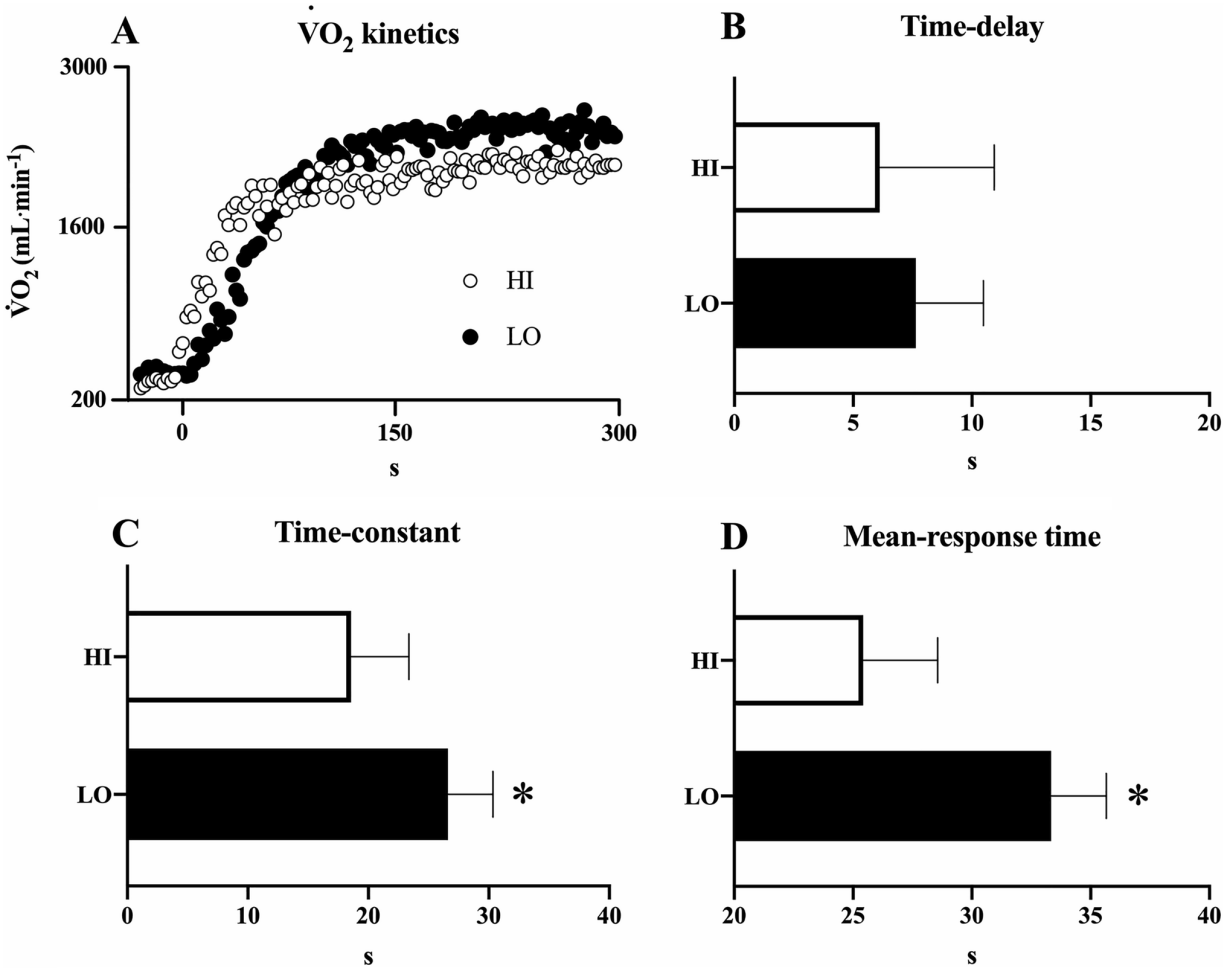
The rate of $\dot{V}$O2 increase at submaximal exercise for both HI and LO. Panel **A** shows the rate of $\dot{V}$O_2_ increase ( $\dot{V}$O_2_ kinetic) for two representative subjects (HI: white circles; LO: black circles). The time-delay (Panel **B**), the time-constant (Panel **C**) and the mean-response time (Panel **D**) are illustrated for each subject (white circles) in HI (white bar) and LO (dark-grey bar) group. ^#^P < 0.05 vs HI

The $\dot{V}$O_2_ kinetics was *largely* to *very largely* (ES: -2.74/-1.76) faster (*P* > 0.05) in HI than LO: despite *small* [ES:-0.36(CI: -1.35/0.63] non-significant differences (*P* > 0.05) in time-delay, HIGH showed a *large* [ES:-1.76(CI:−2.92/−0.61] and *very-large* [ES:-2.74(−4.10/−1.37)] difference with a faster time-constant and mean-response time than LO, respectively (Fig. 2).

### Between-protocols differences at maximal exercise

As shown in Table 1, V_max_ was *largely* higher in CP1 *vs* DP for both HI [*P* < 0.001, ES:1.96(0.77/3.16)] and LO [*P* < 0.001, ES: 1.84(0.67/3.01)]. In CP1 *vs* CP2, V_max_ was *largely* higher for HI [*P* < 0.001, ES: 1.73, CI: 0.58/2.88)] and *moderately* higher for LO [*P* = 0.006, ES: 1.11(0.06/2.17]. In CP2 *vs* DP, V_max_ was *moderately* higher for LO [*P* = 0.039, ES: 0.75(−0.26/1.76)], while *small* not significant V_max_-difference for HI [*P* = 0.102, ES: 0.30(−0.68/1.29)] were retrieved.

No between-protocol (CP1 vs CP2 vs DP) differences for maximum $\dot{V}$O_2_, VCO_2_, RER, SaO_2_, *f*_H_, VE and BLa^−^_peak_ were found for both HI and LO. Similarly, no between-protocol differences in general-, respiratory- and muscular-RPE were found.

### Between-protocols differences at submaximal exercise

As shown in Fig. 1, $\dot{V}$O_2_/velocity slope showed a *moderate* difference in CP1 *vs* DP for HI [*P* = 0.003, ES:−0.85(−1.88/−0.17)] and a *large* difference for LO [*P* = 0.002, ES: −1.75(−2.91/−0.60)]. In CP1 *vs* CP2, $\dot{V}$O_2_/velocity slope showed a *small* difference for HI [*P* = 0.003, ES:−.48(−1.48/0.51)] and *very large* difference for LO [*P* = 0.007, ES: -5.97(−8.26/-3.68)]. In CP2 vs DP, $\dot{V}$O_2_/velocity slope showed a *trivial* no-significant difference for HI [*P* = 0.283, ES: −0.20(−1.18/0.79)] and a *very large* difference for LO [*P* = 0.016, ES: −2.33(−3.60/−1.06)].

In CP1 *vs* DP, $\dot{V}$O_2_/velocity intercept showed a *small* difference for HI [*P* < 0.001, ES:0.21(−0.78/1.19)] and a *moderate* difference for LO [*P* = 0.002, ES: 0.99(0.05/2.03)]. In CP1 *vs* CP2, $\dot{V}$O_2_/velocity intercept showed a *trivial* difference for HI [*P* = 0.010, ES:0.10(-0.88/1.08)] and a *moderate* difference for LO [*P* = 0.015, ES:0.61 (-0.36/1.60)]. In CP2 vs DP, $\dot{V}$O_2_/velocity intercept showed a *trivial* no-significant differences for HI [*P* = 0.348, ES: 0.00(−0.98/0.98)] and a *very large* difference for LO [*P* < 0.001, ES: 1.51(0.40/2.62)].

### ***Between-protocol V***_***max***_*** correlations***

*Very large* between-protocol correlations for V_max_ were calculated for HI (*r* = 0.73, *r* = 0.84, and *r* = 0.73 for CP1 *vs* DP, CP2 *vs* DP and CP1 *vs* CP2, respectively *P* < 0.05). *Moderate* to *large* between-protocol correlations for V_max_ were calculated for LO (*r* = 0.49, *r* = 0.68, and *r* = 0.79 for CP1 *vs* DP, CP2 *vs* DP and CP1 *vs* CP2, respectively *P* < 0.05).

### Relationship between training status and between-protocol differences

The percentage of the V_max_ in CP1 *vs* DP showed a *small* [*P* = 0.045, ES: -0.53 (-1.56/0.46)] difference between HI and LO [+ 13.3(5.4)% and + 17.9(10.2)%,, respectively] and a *moderate* [*P* = 0.032, ES:−0.75 (−1.76/0.26)] difference for CP2 *vs* DP [+ 6.2(6.6) and + 2.1(3.7)% for HI and LO, respectively].

**Fig. 3:**
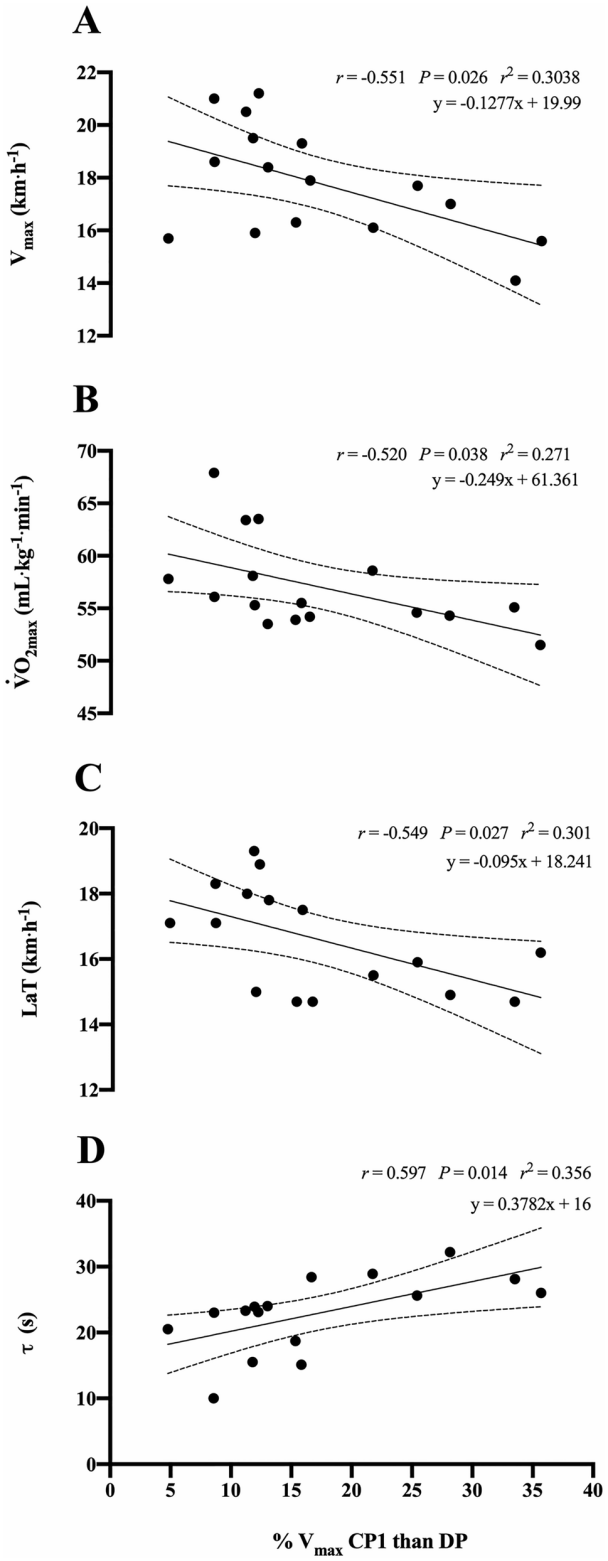
Relationship between training status and the between-protocol V_max_ difference. The percentage of the individual V_max_-difference in CP1 than DP is related with the velocity associated with maximum oxygen uptake (V_max_, Panel **A**), maximum oxygen uptake ( $\dot{V}$O_2max_, Panel **B**) and lactate threshold (LaT, Panel **C**). Regression equations (y = a · bx), 95% confidence intervals and correlation coefficients are also reported

As shown in Fig. 3, the percentage of the V_max_-difference in CP1 than DP showed an inversely *large* correlation with V_max_, $\dot{V}$O_2max_ and the velocity at lactate threshold. Conversely, the percentage of the V_max_-difference in CP1 than DP was *largely* correlated with the time-constant of the $\dot{V}$O_2_ kinetics.

## Discussion

The main finding of the present study was that HI, with faster $\dot{V}$O_2_ kinetics, had lower differences in V_max_ between CP and DP than LO. This observation may confirm the experimental hypothesis stating that athletes with higher aerobic capacity and faster $\dot{V}$O_2_ kinetics are able to adjust better to work rate increments typical of CP with short stage duration. Noticeably, HI had a similar V_max_ in DP and CP2 (i.e., the continuous protocol with slower work rate increments) and the difference in V_max_ between CP1 and DP was lower than in LO. Lastly, the percentage of the V_max_ differences between CP1 and DP were inversely correlated with V_max_, $\dot{V}$O_2max_ and directly correlated to the time-constant of the $\dot{V}$O_2_ kinetics, providing further evidence that between-protocol V_max_ differences in HI are minimized likely because of their faster $\dot{V}$O_2_ kinetics.

### Preliminary considerations

The present results came with no between-protocol differences in $\dot{V}$O_2max_ and in the other main cardiorespiratory and metabolic parameters in both HI and LO. Despite some previous findings about the effects of protocol (i.e. workload *vs* recovery ratio) on $\dot{V}$O_2max_ (Vinetti et al. 2017), these findings reinforce previous data demonstrating that $\dot{V}$O_2max_ was independent of the protocol adopted across different incremental testing procedures (Bentley et al. 2007; Billat et al. 1996; Riboli et al. 2017). The present outcomes are in line with previous literature, in which no differences in $\dot{V}$O_2max_ were observed between protocols in different populations, such as recreationally-active men (Kirkeberg et al. 2011), physically-active young adults (Riboli et al. 2017), semi-professional soccer players (Riboli et al. 2021) and competitive middle- and long-distance runners (Billat et al. 1996; Kuipers et al. 2003). Similar results were also found in moderately-active cyclists during cycle-ergometric evaluation (Adami et al. 2013).

### Maximum exercise

The present findings demonstrate that V_max_ was protocol-dependent, as also previously observed (Kuipers et al. 2003; Riboli et al. 2017, 2021). The steeper the work rate increase, the higher the V_max_ in both groups. In LO V_max_ differed in each protocol (i.e., CP1 > CP2 > DP). Conversely, in HI the V_max_ differences between CP2 and DP were not present (i.e., CP1 > CP2 = DP). These findings suggest that higher aerobic capacity may minimize the between-protocol V_max_ differences due to the faster cardiorespiratory and metabolic adjustments to match the increasing mechanical power in CP. This explanation was further supported by the faster $\dot{V}$O_2_ kinetics in HI, in which no difference was found between CP2 and DP. On the contrary, in LO V_max_ in CP2 was higher than in DP due to the slower $\dot{V}$O_2_ kinetics. A direct comparison with previous studies is challenging, as this was the first study investigating the effect of aerobic training status on V_max_. Previous studies observed a greater between-protocol difference when steeper work rate vs time increments were utilized (Kuipers et al. 2003; Riboli et al. 2017, 2021). Indeed, when comparing three CPs with 1-, 3- or 6-min stage duration in competitive middle-distance runners, V_max_ was related to the slope of the work rate *vs* velocity increments (Kuipers et al. 2003). Similar results were found when a CP with different work rate *vs* velocity increments was used during cycle ergometry in active people (Adami et al. 2013) or international competitive triathletes (Bentley and McNaughton 2003). Recently, greater peak mechanical power output was found also in healthy participants using a synchronous arm crank ergometry when work rate increments were steeper (Kouwijzer et al. 2019). Interestingly, when long-distance runners were tested using CP with different stage duration but similar slope in the velocity *vs* time increments (e.g., 1 km·h^−1^ increments every 2 min *vs* 0.5 km·h^−1^ increments every min), no difference in V_max_ was detected (Billat et al. 1996). Similar findings were observed also in sedentary men on cycle ergometer (Zhang et al. 1991).

### Submaximal exercise

A faster $\dot{V}$0_2_ kinetics was observed in HI than in LO participants during the test at 10 km/h, implying a more rapid cardiorespiratory and metabolic adjustment capacity to match mechanical power increase during incremental exercise. Previous investigations observed that athletes with a high aerobic capacity, such as long- and middle-distance runners, were qualified by greater physiological characteristics in terms of faster $\dot{V}$O_2_ kinetics (Poole and Jones 2012; Coyle 1995). In top-level aerobic athletes, indeed, an extremely short time (i.e., ~ 30 to ~ 40 s) is required to achieve a $\dot{V}$O_2_ steady-state (Poole and Jones 2012), while in trained healthy individuals at least 2–3 min or even more are required (Robergs 2014; Poole and Jones 2012). The present results confirm the current hypothesis demonstrating a lower between-protocol V_max_ difference in HI than in LO likely due to the changes in running velocity faster than cardiorespiratory and metabolic adjustments. This was remarkably highlighted by no-differences in V_max_ between CP2 and DP for HI.

The between-protocol difference in the $\dot{V}$O_2_/velocity slope, was greater in LO (*large* to *very large*) than in HI (*trivial* to *moderate*), leading the slope to CP1 > CP2 > DP and CP1 > CP2 = DP in LO and HI, respectively. High-level aerobic athletes are also qualified by better biomechanical characteristics matching with a faster $\dot{V}$O kinetics and a higher running economy (Coyle 1995). In the present study, LO showed a reduced $\dot{V}$O_2_/velocity slope in both CP1 and CP2 than DP, while in HI the difference between CP2 and DP disappeared. This condition typically occurs when the time to reach cardiorespiratory and metabolic equilibrium matches the change in work rate across CPs.

### Training status and between-protocol differences

The between-protocol V_max_ differences were inversely correlated with training status. A higher $\dot{V}$O_2max_, V_max_, lactate threshold and faster $\dot{V}$O_2_ kinetics provided further evidence that between-protocol V_max_ differences in HI may be likely counteracted by their higher aerobic training status. Therefore, a more consistent V_max_ across different protocols in athletes with a higher aerobic capacity was found. The knowledge of the between-protocols V_max_ differences could have practical implications for testing, exercise prescriptions and physiological outcomes during running activities. Different % V_max_ were shown to lead different physiological responses by increasing or decreasing the time spent at ~  $\dot{V}$O_2max_, a crucial factor for chronic adaptations and performance development (Buchheit and Laursen 2013). Therefore, a more consistent V_max_ determination should permit a more accurate running exercise prescription in both HI and LO athletes.

### Methodological considerations

Some methodological considerations should accompany the present investigation. First, the study of the dynamic response of metabolic and pulmonary variables upon exercise onset is strongly affected by the recording technique (Ferretti 2015). The Auchincloss algorithm (Auchincloss et al. 1966) utilized to calculate dynamic $\dot{V}$O_2_ responses requires a correct determination of the change in the amount of gas stored in the lungs over each breath. However, the algorithm estimated the end-expiratory lung volume imposing fixed pre-defined values of end-expiratory lung volumes (Ferretti 2015) leading to an impossibility of attaining a correct estimation (di Prampero and Lafortuna 1989). Subsequently, it was demonstrated a two-time improvement of the signal-to-noise ratio in breath-by-breath alveolar gas transfer (Capelli et al. 2001) and a lower dynamic response (Cautero et al. 2002) using Grønlund algorithm. However, despite such algorithm improvements, the aforementioned issue could not be fixed (Ferretti 2015). Secondly, despite a stepwise interpolation procedure was proposed to improve the time-constant calculation (Lamarra et al. 1987), a slightly higher time-constant than the interpolation interval still remains. Therefore, at least in the light exercise domain, mere stacking of multiple repetitions was proposed if the data were from the same $\dot{V}$O_2_ on rest-to-exercise transient (Bringard et al. 2014; Francescato et al. 2014b, a). As such, attempts at improving the time resolution beyond the single-breath duration could rely only on computational manipulations, such as superimposition of several trials and interpolation procedures (Francescato et al. 2014a; Francescato and Cettolo 2020).

Lastly, the present findings open to new future perspectives. During submaximal running bouts, the time shift between velocity and $\dot{V}$O_2_ could be calculated knowing the time constant of the $\dot{V}$O_2_-on kinetics. Therefore, a mathematical modeling would possibly provide a calibration equation for V_max_ correction in CP1 and CP2 with respect to DP.

### Practical considerations

The between-protocol V_max_ differences in CP1 (+ 18% and + 13% than DP in LO and HI, respectively) and CP2 (+ 6% than DP in LO) should be considered for both athletes aerobic profiling and exercise prescription. These results suggest that in LO a protocol with more than 2 min stage durations is required for the metabolic power to match the mechanical power. In HI, a 2-min stage duration may be suitable and can be consistently utilized within sport contexts. When shorter stage durations are mandatorily required (e.g., 1-min), a misestimate V_max_ should be considered to plan accurately high-intensity exercises in both HI and LO. Indeed, different %- V_max_ are suggested to increase the time spent at ~  $\dot{V}$O_2max_ during high-intensity interval or intermittent exercises (e.g., 110% to 130%-V_max_ for short-intervals exercises or 130% to 160%-V_max_ for repeated sprint trainings) (Buchheit and Laursen 2013). Therefore, when short intervals exercises (e.g., ~ 110% V_max_) are prescribed, ~ 18% of V_max_ difference in CP1 *vs* DP for LO should induce an unexpected greater anaerobic involvement leading to acute physiological responses similar to a running exercise at ~ 130%-V_max_ (i.e., ~ 25 km·h^−1^ instead of ~ 21 km·h^−1^). Similar differences between desired and actual physiological responses could be found across any %-V_max_ within both longer and shorter running exercises. Neglected between-protocol V_max_ differences may mislead acute physiological responses (e.g., more aerobic or anaerobic contribution) and possibly negatively affect the training adaptations, especially within-athletes with lower training status. Therefore, the knowledge of the between-protocol differences may help practitioners to properly manage different testing modalities and to adjust the %-V_max_ when intermittent or interval running-based exercises are prescribed.

### Conclusions

As previously observed, CP and DP can be used interchangeably to assess $\dot{V}$O_2max_, but not V_max_ (Riboli et al. 2017, 2021). We demonstrate here that aerobic training status can influence the magnitude of the between-protocol differences in V_max_ assessment. When different protocols are utilized to determine V_max_, between-protocol differences exist, especially in CPs *vs* DP in which a matching between metabolic and mechanical power clearly occurs. These V_max_ differences should be considered when athletes with different aerobic training status are tested. The V_max_ difference between CPs and DP disappeared in HI during CP2, suggesting that a protocol with at least 2-min stage duration may be sensitive enough in athletes with a greater aerobic capacity, while differences still exist across participants with lower aerobic training status for which at least 3-min stage duration seems required. These between-protocol V_max_ differences should be considered when athletes with different aerobic capacity are tested because they may affect the testing outcomes and training prescriptions.

## Data Availability

Data and materials are available on request to the corresponding author.

## Abbreviations

HI: Group with high $\dot{V}$O_2max_
LO: Group with low $\dot{V}$O_2max_
CP1: Continuous incremental protocol [1 km·h-1 per min]
CP2: Continuous incremental protocol [1 km·h-1 every 2 min]
DP: Discontinuous incremental protocol
$\dot{V}$O_2max_: Maximum oxygen uptake
$\dot{V}$O_2_/Velocity slope: Regression analysis of the $\dot{V}$O_2_ vs velocity relationship at submaximal workloads
$\dot{V}$O_2_ kinetics: $\dot{V}$O_2_-transition from rest to steady-condition
Vmax: The velocity associated with maximum oxygen uptake
$\dot{V}$CO_2_: Carbon dioxide production
RER: Respiratory exchange ratio
SaO_2_: Arterial O_2_ saturation
$$\dot{VE}$$: Expiratory ventilation
BLa-: Blood lactate concentration
RPE: Rate of perceived exertion
ANOVA: Analysis of variance
ES: Effect size
95% CI: 95% Confidence intervals

## Code availability

Protocol #102/14.
